# Magnesium activation affects the properties and phosphate sorption capacity of poultry litter biochar

**DOI:** 10.1007/s42773-023-00263-5

**Published:** 2023-10-07

**Authors:** Joshua T. Padilla, Donald W. Watts, Jeffrey M. Novak, Vasile Cerven, James A. Ippolito, Ariel A. Szogi, Mark G. Johnson

**Affiliations:** 1 United States Department of Agriculture, Agricultural Research Service, Coastal Plains Soil, Water and Plant Research Center, Florence, SC 29501, USA.; 2 School of Environment and Natural Resources, The Ohio State University, Columbus, OH 43210, USA.; 3 Center for Public Health and Environmental Assessment, United States Environmental Protection Agency, Corvallis, OR 97331, USA.

## Abstract

Biochars with a high affinity for phosphorus (P) are promising soil amendments for reducing P in agricultural run-off. Poultry litter (PL) is an abundant biochar feedstock. However, PL-derived biochars are typically high in soluble P and therefore require chemical modification to become effective P sorbents. This study investigated the effect of magnesium (Mg) activation on extractable P (EP) and P sorption capacities of PL-derived biochars. Biochar was produced at 500–900 °C from PL activated with 0–1 M Mg. Three differentially aged PL feedstocks were evaluated (1-, 3–5-, and 7–9-year-old). Increased Mg activation level and pyrolysis temperature both resulted in EP reductions from the biochars. Specifically, biochars produced at temperatures ≥ 700 °C from PL activated with ≥ 0.25 M Mg had negligible EP. X-ray diffractograms indicated that increased Mg loading favored the formation of stable Mg_3_(PO_4_)_2_ phases while increasing temperature favored the formation of both Mg_3_(PO_4_)_2_ and Ca_5_(PO_4_)_3_OH. Maximum P sorption capacities (P_max_) of the biochars were estimated by fitting Langmuir isotherms to batch sorption data and ranged from 0.66–10.35 mg g^−1^. Average P_max_ values were not affected by PL age or pyrolysis temperature; however, biochars produced from 1 M Mg-activated PL did have significantly higher average P_max_ values (*p* < 0.05), likely due to a greater abundance of MgO. Overall, the results demonstrated that Mg activation is an effective strategy for producing PL-derived biochars with the potential ability to reduce P loading into environmentally sensitive ecosystems.

## 1 Introduction

The commercial poultry industry produces 0.7–2.0 tons of poultry litter (PL) per 1000 broilers (Bolan et al. 2010). For the United States, this currently amounts to an estimated 6.4–18.3 million tons of PL produced per year from broiler production (USDA 2022). Poultry litter is a nutrient-rich material commonly applied in agricultural settings as a nitrogen, potassium, and phosphorus (P) fertilizer (Ashworth et al. 2019). However, the over-application of PL can result in the accumulation of soil P exceeding plant nutritional requirements, commonly referred to as “legacy P” (Bryant et al. 2022). Legacy P can serve as a persistent source of nutrient loading into water bodies receiving agricultural runoff, and is therefore considered an environmental risk. For example, widespread eutrophication in the Chesapeake Bay has been partially attributed to legacy P soil losses from agricultural areas of the Delmarva Peninsula due to PL over-application (Vadas et al. 2018; Kleinman et al. 2019). Legacy P losses pose a similar risk to water quality in several other geographical areas (Motew et al. 2017; Dari et al. 2018; Van Meter et al. 2021). As such, strategies to manage legacy P are needed.

One such strategy is the amendment of soils with biochar that has a high affinity for soluble P. For instance, the application of corncob- and rice husk-derived biochar at a rate of 1% (by wt.) increased the P sorption capacity of a neutral pH soil by a factor of 1.7 and 1.9, respectively (Eduah et al. 2019). Similarly, Xu et al. (2014) demonstrated that the application of wheat straw biochar (up to 10% by wt.) increased the P sorption capacity of two acidic soils by a factor of 1.4–1.9, while Chintala et al. (2014) found that corn stover and switchgrass biochars (4% by wt.) significantly increased the P sorption capacity of an alkaline soil. Others reported similar results for a variety of contrasting soils (Morales et al. 2013; Dari et al. 2016; Ngatia et al. 2017; Cerven et al. 2021), demonstrating that biochar application may be a viable approach for controlling legacy P mobility in soils.

A plausible approach for controlling legacy P mobility in soils, within the context of PL land applications, is the creation and use of PL-derived biochars. Poultry litter is an abundantly used soil amendment where broiler chickens are raised, but PL has a high soluble P content (Wang et al. 2015) that has led to soil issues associated with legacy P (Feyereisen et al. 2010; Bryant et al. 2022). If there were a means by which PL could provide some necessary nutrients for plant growth, yet sorb excess legacy P created from historic PL land application, this could lead to a win–win for the poultry industry and the environment.

The sorption of P by pristine biochar is typically low (Vikrant et al. 2018), and therefore, chemical modifications to biochar to improve its P sorption capacity have received considerable attention. The pre- or post-pyrolysis impregnation of biochar with metals such as Al, Fe, Ca, and Mg as their chloride salts has been demonstrated to increase biochar’s affinity for P (Vikrant et al. 2018; Yang et al. 2019; Zhang et al. 2020; Bian et al. 2023). However, from a practical standpoint, Mg is the most cost-effective of the metal chlorides when producing activated biochars at scale (Ding et al. 2019), and Mg is more environmentally friendly and less toxic to plants than other metals such as Al (Imadi et al. 2016).

Producing an effective P sorbent from PL-derived biochar is a unique challenge due to its high soluble P content (Wang et al. 2016). The Mg activation of PL prior to pyrolysis could be a novel solution to this problem because this approach has been shown to increase the P sorption capacity of biochars produced from a wide range of other feedstocks. For example, Shin et al. (2020) reported that Mg activation of a ground coffee waste biochar increased the P sorption capacity from negligible to 56 mg P g^−1^, attributed to the formation of Mg-P mineral surface precipitates. Likewise, biochars produced from Mg-activated carrot residues had a maximum P sorption capacity of 130 mg g^−1^, whereas unactivated biochars were incapable of sorbing P (Pinto et al. 2019). Mg activation has been demonstrated to increase the P sorption capacity of biochars in several other cases (Takaya et al. 2016; Haddad et al. 2018; Chen et al. 2018; He et al. 2022). As such, activating PL feedstock with Mg before pyrolysis is a promising strategy to convert PL-derived biochar from a net P source to sink.

In general, Mg activation is achieved by soaking the feedstock in a > 1 M MgCl_2_ solution prior to pyrolysis (Takaya et al. 2016; Jiang et al. 2018; Pinto et al. 2019; Shin et al. 2020). From a practical standpoint, using the least amount of MgCl_2_ to achieve the desired P sorption properties would be ideal. However, the efficacy of Mg activation across various Mg concentrations is still unknown. Moreover, it is well known that feedstock properties have a strong influence on the properties of the resulting biochar. The properties of PL have been shown to depend on age (Oyewumi and Schreiber 2012; Temple et al. 2014), and therefore it is expected that PL derived from the same source but varying in age would produce biochar with different properties. Pyrolysis conditions, particularly temperature, have also been shown to affect the P sorption capacity of various biochars; increasing pyrolysis temperatures typically are associated with greater P sorption (Haddad et al. 2018; Jiang et al. 2018). As such, it is also essential to consider a range of pyrolysis temperatures.

We hypothesized that the pre-pyrolysis Mg activation of the PL feedstock would both fix the natural P content of PL-derived biochars and enable them to sorb additional P. We investigated the effect of PL age, pyrolysis temperature, and activating solution Mg concentration on the resulting biochars’ extractable P content, P sorption capacity, and physicochemical properties.

## 2 Materials and methods

### 2.1 Biochar production

Poultry litter (PL) was obtained from active poultry operations in Wicomico County, Maryland, USA. Three different PL feedstocks, based on age, were obtained. One-year-old PL (PL1) was collected during a poultry rearing house cleanout. Poultry litter aged for either 3–5 years (PL3) or 7–9 years (PL7) was collected from covered storage sites. Air-dried PL was passed through a 6.4-mm sieve to remove large debris before further processing. The feedstocks were activated by combining PL and MgCl_2_ solution at a ratio of 1:5 m/v (Chen et al. 2018). Concentrations of the MgCl_2_ (MgCl_2_⋅6H_2_O) solution were either 0, 0.25, 0.5 or 1 M. Poultry litter was soaked in the MgCl_2_ solutions for 48 h, after which the remaining MgCl_2_ solution was drained, and the activated PL was allowed to air-dry. Feedstocks were then pyrolyzed at either 500, 700, or 900 °C in a programable Lindberg furnace equipped with a retort and under a steady flow of N_2_ gas. The temperature of the furnace was increased to 200 °C and held for 1 h, after which the temperature was increased to the final pyrolysis temperature and held for 4 h (Cantrell and Martin 2012). Unactivated biochars were produced by pyrolyzing air-dried PL (without MgCl_2_ solution soaking) at either 500, 700, or 900 °C. In total, 36 biochars were produced representing three feedstocks, four Mg activation levels, and three pyrolysis temperatures.

### 2.2 P sorption isotherms

Phosphorus sorption isotherms for each biochar were obtained by a batch equilibration method. Specifically, 0.1 g of biochar was reacted with 20 mL of solution containing 25–150 mg P L^−1^ in 50 mL polypropylene centrifuge tubes. Input solutions were prepared by dissolving KH_2_PO_4_ in a background of 10 mM KCl. Extractable P from biochars was determined by reacting 0.1 g of biochar with 20 mL of 10 mM KCl with no added P in solution. The input solutions had an average pH of 5.0. Biochar/solution mixtures were transferred to a platform shaker and were continuously shaken at 21 °C for 24 h. Preliminary experiments demonstrated that 24 h of reaction time was sufficient to achieve equilibrium (Additional file 1: Fig. S1). Following shaking, 10 mL of the supernatant was filtered through a 0.45 μm nylon filter and solution P concentrations were determined using inductively coupled plasma-atomic emission spectroscopy (ICP-AES; Agilent 5110, Agilent Technologies Inc.). The effect of pH on P sorption was determined in an identical manner as above with an initial P concentration of 75 mg L^−1^ and an initial solution pH ranging from 4.0 to 8.0. Solution pH was adjusted by adding small volumes of HCl or KOH. The final pH of the supernatant was measured following 24 h of reaction time. The sorbed concentration of added P was determined by the difference between the initial and final P solution concentrations. All experiments were completed in triplicate.

Sorption maximum is a common and widely reported metric for characterizing the affinity of a biochar for P (Luo et al. 2023). The **s**orption maximum for each biochar was estimated by fitting the linear Langmuir isotherm to the observed data according to (Yan et al. 2018):

$$\frac{C}{S}=\frac{1}{KP_{max}}+\frac{C}{P_{max}},$$

where *C* (mg L^−1^) and *S* (mg g^−1^) are the solution and sorbed P concentrations, respectively, *K* is an empirical affinity constant (L mg^−1^), and *P*_*max*_ is the maximum P sorption capacity (mg g^−1^). Sorption data were also described using the linear Freundlich isotherm, given by (Yan et al. 2018):

$$log(S)=\text{nlog}(C)+log\left(K_{f}\right),$$

where *K*_*f*_ is the Freundlich distribution coefficient (mg^1−n^ L^n^ g^−1^) and *n* is a unitless nonlinearity parameter.

### 2.3 Biochar characterization

Biochar total macro- and micronutrient contents were determined by the Clemson University Agricultural Service Laboratory. Specifically, samples were digested using nitric acid and hydrogen peroxide according to the method of Jones and Case (1990) followed by analysis via ICP-AES. Brunauer–Emmett–Teller (BET) surface area was determined via N_2_ adsorption–desorption using a NOVA 2200e Surface Area and Pore Size Analyzer (Quantachrome Instruments). X-ray diffraction (XRD) patterns for each biochar were obtained by analyzing ground (0.25 mm) biochar samples on an Olympus Terra field-portable XRD equipped with a 10 W X-ray tube and Cu target. Data were collected from 5–55° 2θ in 0.02° 2θ steps and diffraction patterns were analyzed using XPowder software to identify diffractogram peaks. Ultimate and proximate analyses of the PL feedstocks and biochars were conducted by Hazen Research, Inc. (Golden, CO, USA) according to ASTM methods D3176 and D3172 (ASTM 2021a; b). Scanning electron microscope (SEM) images and energy dispersive spectroscopy (EDS) analysis of biochar surfaces were obtained using a Vega-3 LMU Scanning Electron Microscope (TESCAN, Brno, Czech Republic). The pH of the prepared biochars was measured in 1:2 (v:v) biochar/DI water mixtures that were stirred for 30 min.

### 2.4 Statistical analysis

All statistical analyses were completed using SAS 9.4. Linear regressions between biochar properties and production variables were determined using the PROC GLM procedure. Comparisons between average biochar properties and production variables were made with Tukey or Tukey–Kramer pairwise comparisons at the *p* < 0.05 significance level using PROC GLM.

## 3 Results and discussion

### 3.1 Extractable P

**Fig. 1:**
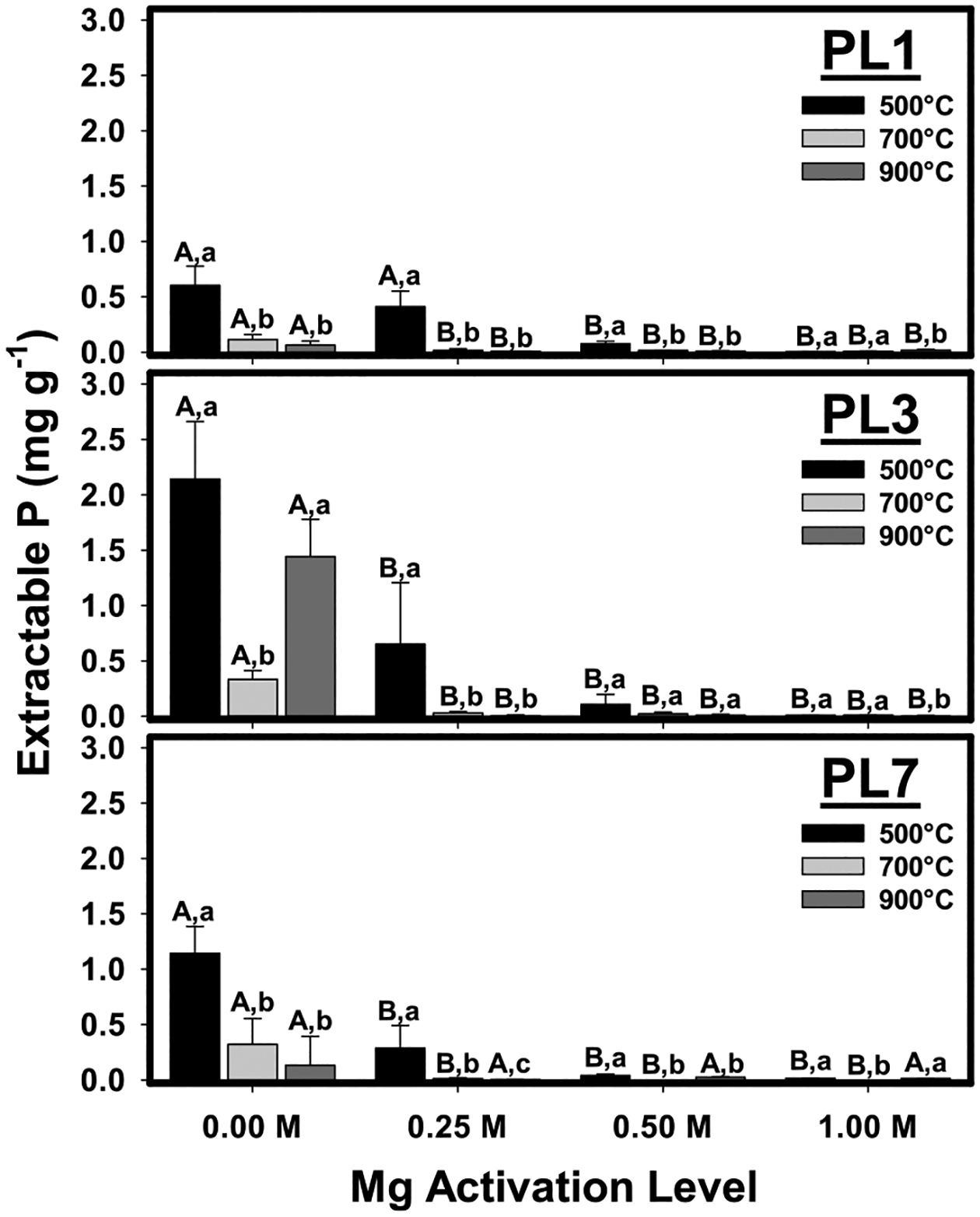
Extractable P from biochars produced from poultry litter aged 1-year (PL1), 3–5 years (PL3), or 7–9 years (PL7) at 500, 700, or 900 °C. Error bars are the 95% confidence intervals. For each feedstock, different uppercase letters indicate significant differences (*p* < 0.05) between Mg activation levels for a given temperature, while different lowercase letters indicate significant differences (*p* < 0.05) between pyrolysis temperatures for a given Mg activation level, as determined by a Tukey pairwise comparison test

Average extractable P (EP) concentrations from each biochar are shown in Fig. 1. Within either PL1, PL3, or PL7, when comparing within an individual pyrolysis temperature, significant differences for EP between Mg activation levels were present (indicated by different uppercase letters in Fig. 1). In general, the activation of the PL feedstock with Mg reduced EP. In nearly all cases, activation using 0.25 M Mg significantly reduced EP relative to unactivated biochars, while further increases in Mg activation level did not result in additional EP reductions. Exceptions to this were biochars produced from PL1 at 500°C and PL7 at 900°C. The former required 0.5 M Mg activation for significant reductions in EP, while no significant differences in EP at any Mg activation level were observed for the latter, likely due to large variability in EP from the unactivated biochar.

Within either PL1, PL3, or PL7, when comparing within an individual Mg-activation level, significant differences for EP between pyrolysis temperatures were present (indicated by different lowercase letters in Fig. 1). Overall, EP tended to decrease with increasing pyrolysis temperatures except for unactivated PL3 biochars. Unactivated PL1 and PL7 biochars produced at 700 °C and 900 °C were similar yet had significantly lower EP concentrations than those produced at 500 °C. For activated biochars, pyrolysis temperatures of 700 °C and 900 °C resulted in nearly negligible EP in all cases.

Comparisons of EP from different poultry litter feedstocks for each combination of pyrolysis temperature and Mg activation level are shown in Additional file 1: Fig. S2. In some cases, significant differences (*p* < 0.05) were observed between the different feedstocks. For instance, for unactivated biochars produced at 500 and 900 °C, EP from PL3 biochars was significantly greater than that from PL1 or PL7 biochars. However, trends were inconsistent and no significant differences between the feedstocks were observed in most cases. Moreover, when holding pyrolysis temperature and/or Mg activation level constant, no significant correlation (*p* > 0.05) was observed between feedstock age and EP, confirming that EP from the biochars was not controlled by the feedstock.

**Fig. 2:**
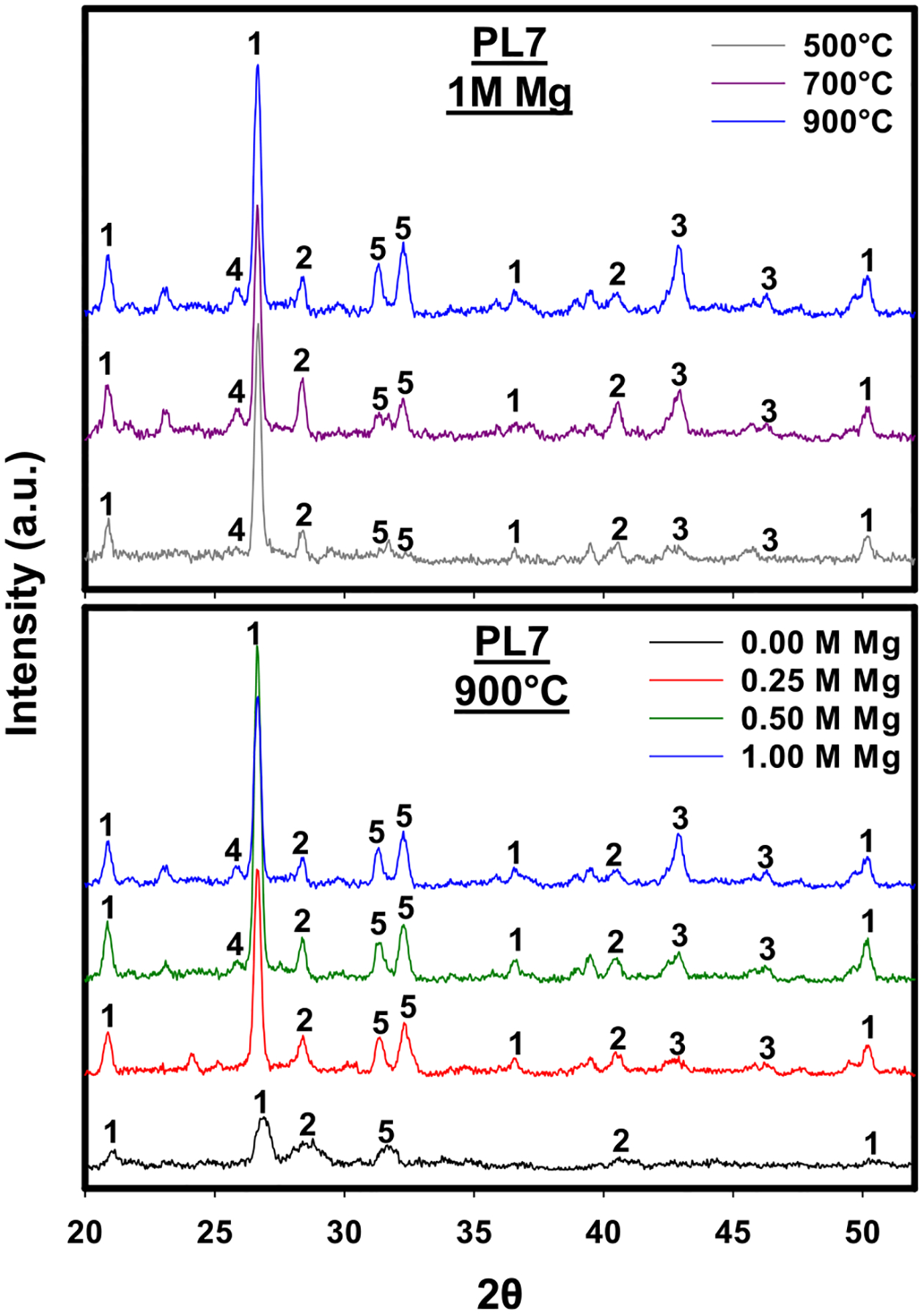
X-ray diffraction patterns for biochar produced from 1 M Mg-activated poultry litter aged for 7–9 years (PL7) at various temperatures (top panel) and PL7 biochars produced at 900°C with varying Mg activation levels (bottom panel). Characteristic peaks are identified as: quartz (1), sylvite (KCl) (2), MgO (3), Mg_3_(PO_4_)_2_ (4), Ca_5_(PO_4_)_3_OH (5)

**Fig. 3:**
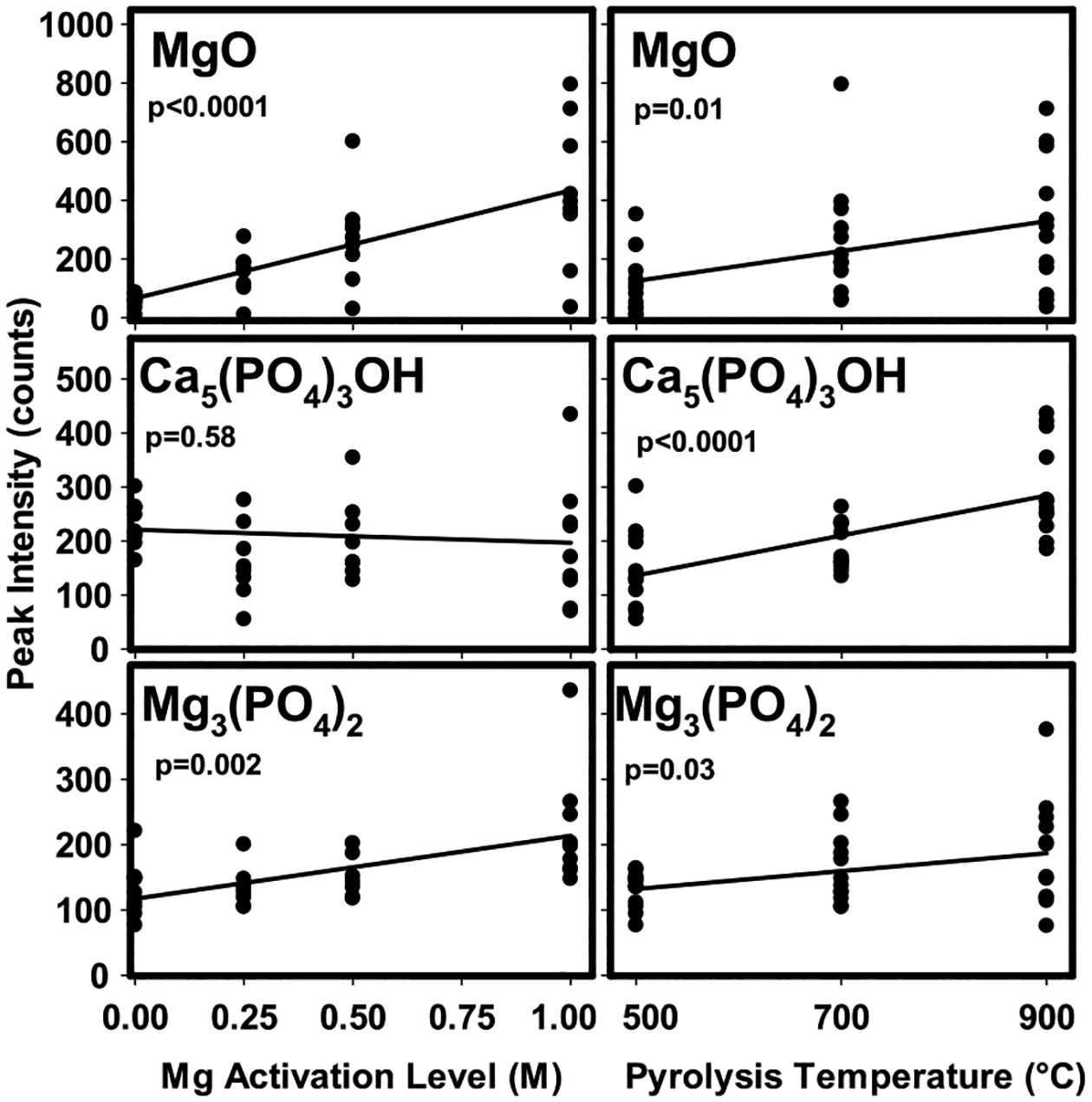
Peak intensities from X-ray diffraction patterns of poultry litter biochars as a function of Mg activation level and pyrolysis temperature. Solid curves are simple linear regressions and p-values for slope estimates are given in the panels. XRD spectra peaks for MgO, Ca_5_(PO_4_)_3_OH, and Mg_3_(PO_4_)_2_ were 42.88 2θ, 31.34 2θ, and 25.80 2θ, respectively

X-ray diffraction patterns for PL7 biochars produced at different temperatures (top panel) and different Mg activation levels (bottom panel) are displayed in Fig. 2. The diffraction patterns shown in Fig. 2 are representative of those obtained from all biochars, as shown in Additional file 1: Fig. S3. The XRD spectra indicate the presence of crystalline phases of quartz, sylvite, MgO, Mg_3_(PO_4_)_2_, and Ca_5_(PO_4_)_3_OH, and were consistent with previous findings for PL and poultry manure-derived biochars (Jiang et al. 2018; Novais et al. 2018). Qualitative estimates of relative abundances of different crystalline phases in the various biochars were made by comparing peak intensities from X-ray diffraction patterns, with results shown in Fig. 3. A positive correlation between Mg activation levels and peak intensities for Mg_3_(PO_4_)_2_ indicates that increased Mg loading favored the formation of stable Mg-P crystalline phases and is consistent with reduced EP from activated biochars. Moreover, positive correlations between pyrolysis temperature and peak intensities for Mg_3_(PO_4_)_2_ and Ca_5_(PO_4_)_3_OH were observed. These results suggest that the formation of P-bearing crystalline phases was favored at higher temperature and is consistent with reductions of EP with increasing pyrolysis temperature. Peak intensities for MgO were also positively correlated with increasing Mg-activation level and pyrolysis temperature. MgO might play an important role in P sorption by being the nucleation site for Mg_3_(PO_4_)_2_ formation or by increasing the positive charge of the biochar surface.

Previous investigations have focused on changes in P speciation during pyrolysis. For example, Uchimiya and Hiradate (2014) found that the dominant inorganic and organic P species in raw PL feedstock were orthophosphate and phytate, respectively, whereas orthophosphate accounted for nearly all P following pyrolysis at temperatures ≥ 500 °C. Based on differential solubility in different extracting solutions, these authors concluded that P speciation was dominated by amorphous rather than crystalline phases of calcium phosphates at temperatures ≥ 650 °C. Likewise, Novais et al. (2018) detected peaks characteristic of Ca_5_(PO_4_)_3_OH in X-ray diffractograms of poultry manure biochar produced at 350°C; however, no such peaks were observed for biochar produced at 650 °C, indicating a decrease in crystallinity at higher temperatures. While these findings contrast with our observations of increased peak intensities with higher temperature, our results were consistent with those of Jiang et al. (2018) where P speciation in PL-derived biochars produced at 600 °C was dominated by crystalline Ca_5_(PO_4_)_3_OH and Mg_3_(PO_4_)_2_. Moreover, Zhang et al. (2021) also reported increasing peak intensities with temperature for Mg_3_(PO_4_)_2_ and Ca_5_(PO_4_)_3_Cl for swine manure-derived biochars produced at 700–900 °C. Inconsistencies in reported relationships between temperature and crystalline phase formation may be due to the heterogeneity of the feedstock materials. For instance, the thermal stability of apatite is dependent on Ca/P ratios (Tõnsuaadu et al. 2012), while materials labeled “poultry litter” have been shown to have five-fold and three-fold differences in Ca and P contents, respectively (Jackson et al. 1975; Felton et al. 2004; Abdala et al. 2012; Novak et al. 2018). As such, the abundance of crystalline P-bearing mineral phases as a function of pyrolysis temperature may be dependent on the specific PL feedstock.

### 3.2 P Sorption

**Fig. 4:**
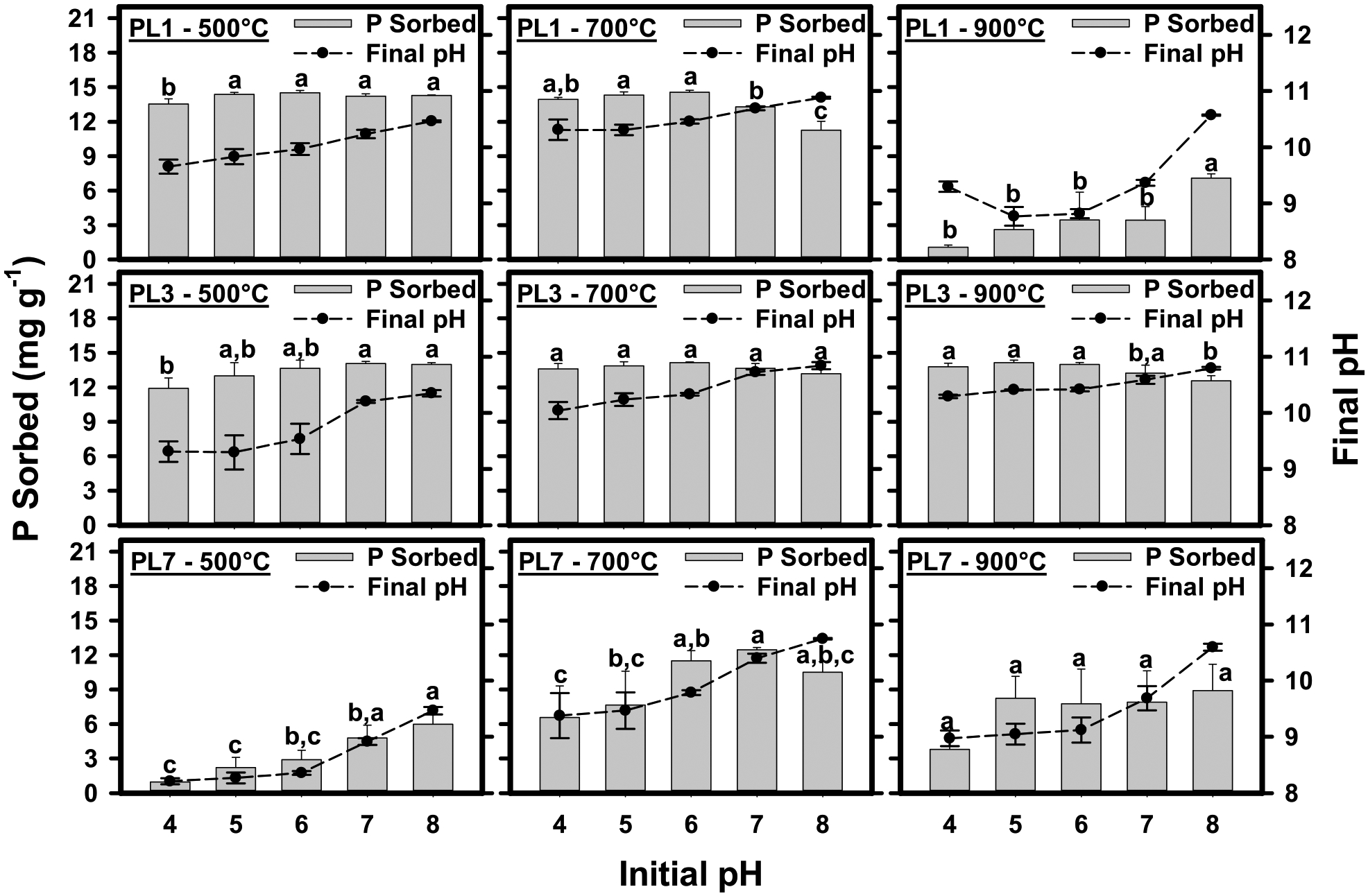
The sorption of P by 1.0 M Mg activated biochars derived from poultry litter aged 1 year (PL1), 3–5 years (PL3), or 7–9 years (PL7) as a function of the initial pH of the solution (Left y-axis). Closed circles show the final pH of the solution (Right y-axis). Error bars are the 95% confidence intervals. For each feedstock and pyrolysis temperature, different letters indicate significant differences in the sorbed P concentration at the p > 0.05 level according to a Tukey pairwise comparison test

Solution pH is one of the most influential experimental variables controlling the sorption of P by biochars due to its effect on surface charge and solution speciation (Luo et al. 2023). The reported effect of pH on P sorption by Mg-activated biochars has been mixed. For example, Shin et al. (2020) observed decreases in average sorbed P concentrations as the pH increased from 4 to 10, a result also found by Jiang et al. (2018). On the other hand, both Pinto et al. (2019) and Haddad et al. (2018) reported increased P sorption as solution pH increased. The sorption of P by 1 M Mg-activated biochars as a function of the initial solution pH is shown in Fig. 4. For each biochar, Tukey pairwise comparisons of final sorbed concentrations for each initial pH were conducted at the *p* < 0.05 significance level. Significant differences are indicated in the figure by different letters. Increasing pH resulted in increased, decreased, or no effect on sorption, depending on the biochar. The pH-dependent sorption of P is controlled by two opposing mechanisms. As pH increases, surface charge decreases and results in the electrostatic repulsion of P in solution, especially as the P anion bears a greater net negative charge at higher pH (Luo et al. 2023). In contrast, the precipitation of Ca- and Mg-P phases is favored at higher pH (Abbona et al. 1982; Pan and Darvell 2009). Therefore, inconsistent trends in P sorption as a function of pH may be due to differences in the relative contribution of the two sorption mechanisms; the former dominates in cases where P sorption decreases with increasing pH, while the latter dominates in cases where P sorption increases. In either case, the formation of Mg-P solid phases likely contributes to P sorption. This is supported by reductions in Mg solution concentrations as pH increases (Additional file 1: Fig. S4), suggesting that Mg is removed from solution by solid phase formation.

**Fig. 5:**
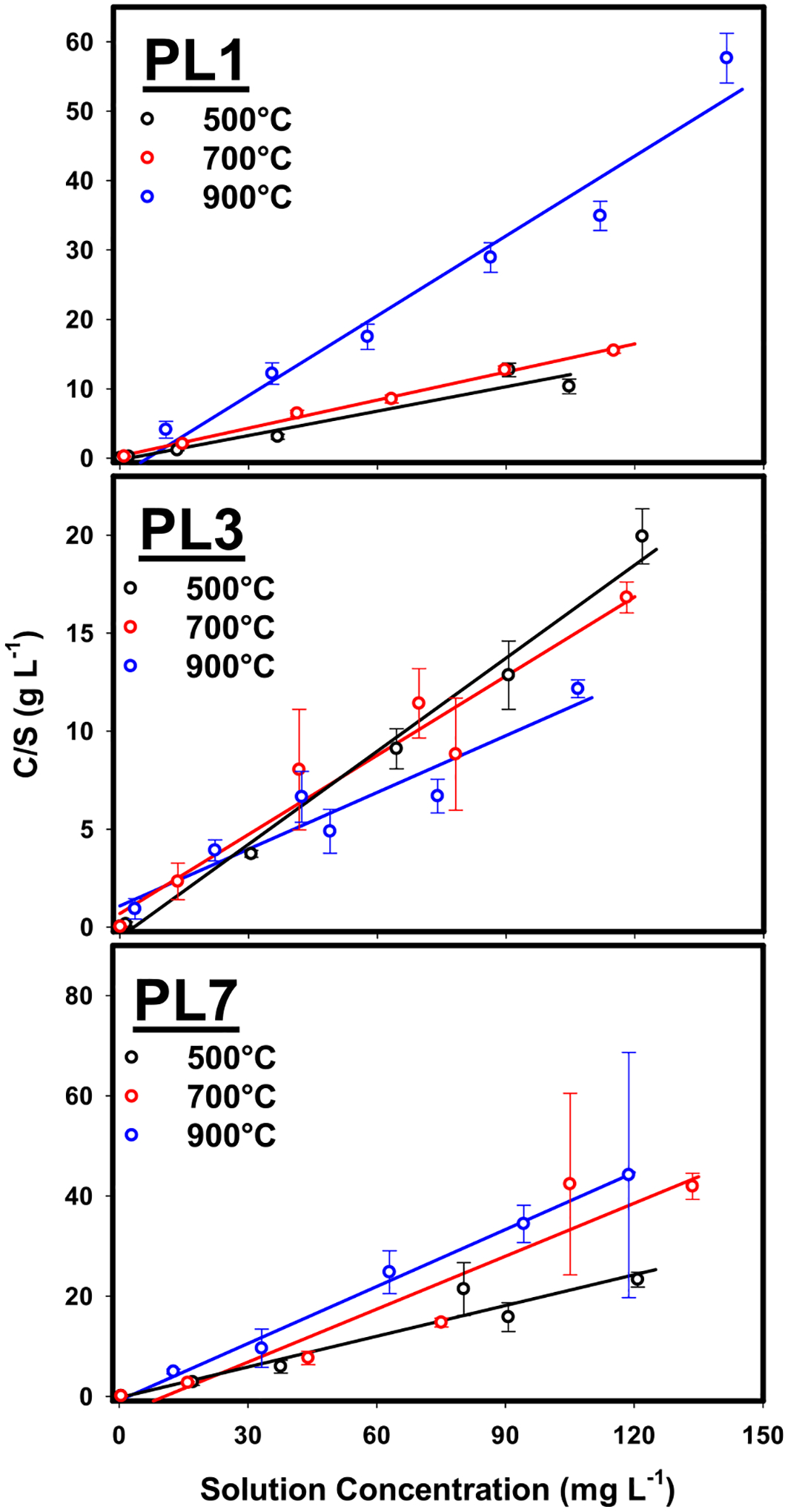
Measured (open symbols) and best-fit Langmuir isotherms (solid curves) for P sorption by 1 M Mg-activated biochars produced from poultry litter aged 1-year (PL1), 3–5 years (PL3), or 7–9 years (PL7) at 500, 700, or 900 °C. Error bars are standard errors of the mean. “C/S” on the y-axis refers to solution P concentration (mg L^−1^) divided by P sorbed concentration (mg g^−1^)

Measured data and best-fit Langmuir isotherms for P sorption (initial pH 5.0) by 1 M Mg-activated biochars are displayed in Fig. 5, while estimates of maximum P sorption capacities (P_max_) for all biochars are given in Table 1. Isotherms for all biochars are shown in Additional file 1: Fig. S5. Several biochars either did not sorb any P or released P into the solution, so estimates of P_max_ were not obtained. These are indicated by “NR” in Table 1. In most cases, unactivated biochars could not remove added P from solution. Unactivated PL3 biochars produced at 700°C and 900 °C did show some ability to sorb P, however, this was inconsistent across the range of input P concentrations and isotherms were poorly described by the Langmuir model (R^2^ < 0.60). Other biochars also displayed inconsistent abilities to sorb P across the range of input P concentrations, so while estimates of P_max_ were obtained, they are likely unreliable as observed data were poorly described. For isotherms that were well described by the Langmuir model (R^2^ > 0.80), values of P_max_ varied from 0.66–10.35 mg g^−1^. The Mg activation of manure-based feedstocks with high contents of P has previously been shown to produce biochars capable of sorbing additional P, consistent with our results. For instance, incorporating Mg-activated cow dung biochar into soil resulted in a nearly ten-fold reduction in leachate P concentrations relative to control soils despite the high P content of the original feedstock (Chen et al. 2018). Others have noted that biochars derived from Mg-activated poultry manure had a P_max_ of up to 250.8 mg g^−1^ (Novais et al. 2018). Best-fit Freundlich isotherm parameters are given in Supplemental Table S1. In most cases, descriptions of the experimental data were poor (R^2^ ≤ 0.57) indicating that the Freundlich isotherm was not appropriate for the current dataset. As such, Langmuir P_max_ values were used as a consistent metric for comparing biochar production variables.

**Fig. 6:**
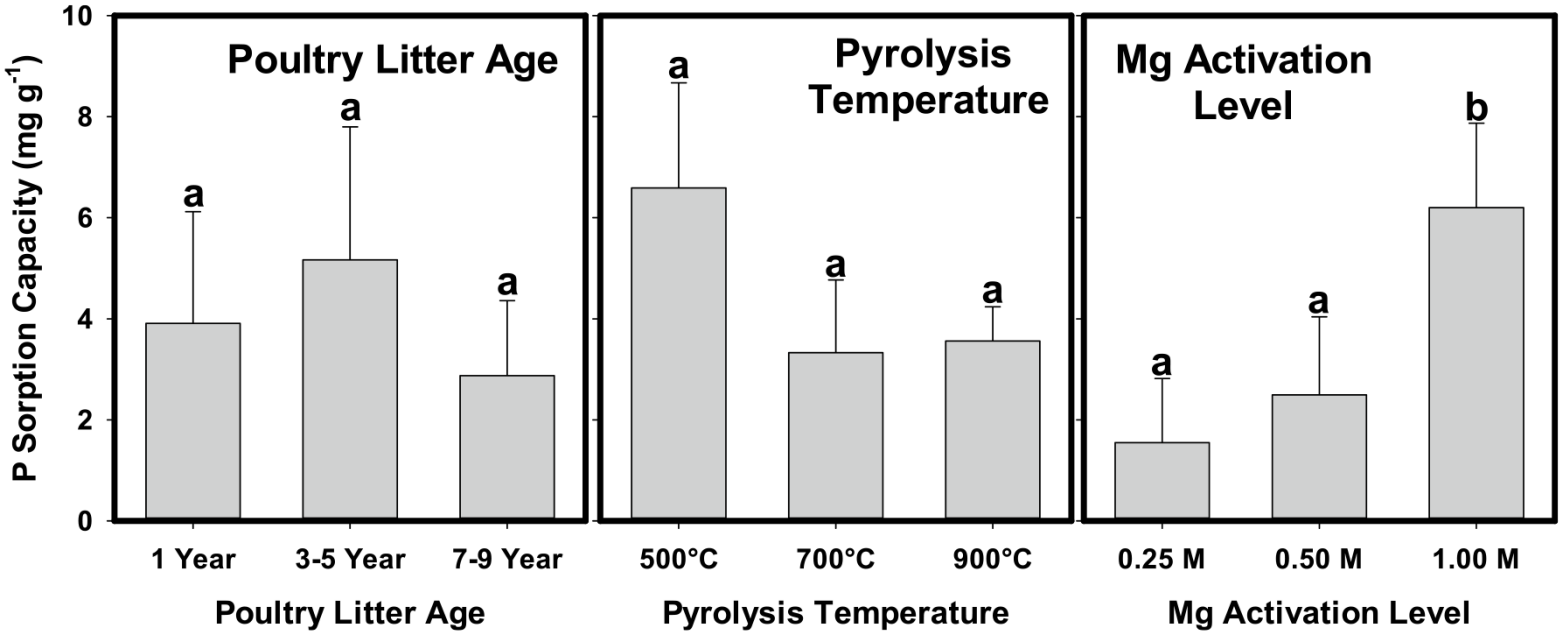
Average estimates of maximum P sorption capacity (P_max_, Table 1) as a function of poultry litter feedstock age, pyrolysis temperature, and Mg activation level. Estimates of maximum sorption capacity were obtained from Langmuir model descriptions of observed isotherm data. Only data that were well described (R^2^ > 0.80, Table 1) were included in the analysis. Error bars are the 95% confidence intervals. Different letters indicate significant differences at the *p* < 0.05 level as determined by a Tukey–Kramer pairwise comparison test

To identify the significant factors that control P_max_, average estimated values as a function of PL age, pyrolysis temperature, and Mg activation level were compared using Tukey–Kramer at the *p* < 0.05 significance level. Biochars that were unable to sorb P or were poorly described by the Langmuir model (R^2^ < 0.80) were excluded from the analysis. The results of this comparison are shown in Fig. 6. No significant differences in average P_max_ were observed for PL age or pyrolysis temperature. A lack of an effect of pyrolysis temperature on the P sorption capacities of Mg-activated biochars contrasts with previous findings. For example, as pyrolysis temperatures increased from 400 to 600 °C, P_max_ values for Mg-activated bamboo and cypress sawdust increased from 344 to 370 mg g^−1^ and 43.4 to 66.7 mg g^−1^, respectively (Jiang et al. 2018; Haddad et al. 2018). On the other hand, Novais et al. (2018) reported that P_max_ for Mg-activated poultry manure biochar decreased as pyrolysis temperature increased from 350 to 650 °C, and Pinto et al. (2019) found P sorption by Mg-activated carrot residues decreased as pyrolysis temperatures increased from 400 to 600 °C. Contrasting evidence of positive, negative, or negligible effects of increasing temperature on P_max_ values for biochars suggests that temperature effects may be largely dependent on the feedstock and any pre-pyrolysis treatments.

Biochars produced from 1 M Mg-activated PL had significantly higher average P_max_ values than those activated with lower Mg levels. This result, coupled with a lack of significant effect of PL age or pyrolysis temperature, indicated that Mg activation level was the most critical control on improving the P sorption capabilities of PL-derived biochars. Several others attributed higher P sorption by Mg-activated biochars to interactions between P and MgO phases. Shin et al. (2020) suggested that the protonation of MgO (MgO + H_2_O ⇋ MgOH^+^ + OH^−^) resulted in a greater positive charge of the biochar surface, increasing the electrostatic attraction of phosphate in solution. Periclase (MgO) bears a net positive charge below pH 9.8 (Kosmulski 2016), so this is a likely possibility. Others have suggested that phosphate precipitation at the surface of MgO results in the formation of Mg-P crystalline or amorphous phases based on SEM–EDS or XRD analyses (Haddad et al. 2018; Pinto et al. 2019; Shin et al. 2020). Based on our XRD analyses, peak intensities for MgO were highly correlated to Mg activation level (*p* < 0.0001, Fig. 3), indicating a greater abundance of MgO with increased Mg loading. Coupled with the findings of others, this suggests that the abundance of MgO accounted for higher average P sorption capacities of the 1 M Mg-activated biochars due to P precipitation with MgO and/or increases in electrostatic attraction between P and the biochar surface.

Sorption of P by hydroxyapatite (HAP; Ca_5_(PO_4_)_3_OH) via surface precipitation has been demonstrated previously (Moreno et al. 1977). Hydroxyapatite was identified in our PL biochars using XRD and may therefore also contribute to the sorption of P in this case. However, HAP was identified in unactivated biochars that were unable to sorb P (Additional file 1: Fig. S3), and total concentrations of Ca were ~2–3 times those of Mg in the same biochars (Additional file 1: Table S2). Moreover, while P_max_ values were related to Mg activation levels, peak intensities for HAP were not (*p* > 0.05, Fig. 3), indicating that Mg activation had no effect on HAP abundance. Collectively, this suggests that MgO was dominant over HAP in controlling the P sorption by the biochars.

Values of P_max_ for biochars produced from the Mg activation of a variety of other feedstocks are shown in Table 2. Sorption capacities ranged from 10.4 mg g^−1^ in the present study to 370 mg g^−1^ for activated bamboo biochar (Jiang et al. 2018). The level of Mg activation varied over an order of magnitude, ranging from 1.4 × 10^–3^–3.3 × 10^–2^ mol Mg per g of feedstock. For practical purposes, activating feedstock with the least amount of Mg to achieve a given sorption capacity is preferred. To assess this, values of P_max_ for each biochar were divided by the ratio of Mg:feedstock mass to provide values of mg of P sorption achieved per mol of Mg used, referred to as “Mg Efficiency” in Table 2. Of the manure-based feedstocks, activating the PL feedstock used in this study was the least efficient on a mol of Mg basis for producing a P sorbent, but was more efficient than activating certain lignocellulosic feedstocks such as sugarcane straw or ground coffee waste.

### 3.3 Biochar characterization

#### 3.3.1 Macro and micronutrient content

Biochar total macro and micronutrient concentrations are presented in Additional file 1: Table S2. Total P contents ranged from 2.2–4.4% by weight and were consistent with values reported previously for PL-derived biochars (Uchimiya and Hiradate 2014; Wang et al. 2015).

No significant correlations were observed between the biochars’ total P content and their EP concentrations, even when holding feedstock or pyrolysis temperature as constant. Others suggested that soluble P from biochar increases as the total P content increases (Kim et al. 2018). However, our results demonstrate that EP concentrations from Mg-activated PL biochars were controlled by the formation of crystalline P-bearing phases rather than by the total P content. Likewise, even when holding feedstock or pyrolysis temperature constant, no significant correlations between biochars’ total P content and their P_max_ values (estimated from Langmuir descriptions of sorption data with R^2^ > 0.8, Table 1) were observed, suggesting that the biochars’ natural P content did not compete with added P for sorption sites. Manure-derived biochars are naturally high in P (Wang et al. 2015), yet our results demonstrated that Mg activation of manure feedstocks can produce biochars that are effective P sorbents despite their high natural P contents, consistent with the results of others (Chen et al. 2018; Novais et al. 2018).

Regardless of pyrolysis temperature or Mg activation level, a significant relationship between PL age and total P content in the resulting biochar was observed; as age increased, biochar total P increased. This age effect was expected as volatile C and N compounds are lost from PL as organic matter is mineralized during aging (Tiquia and Tam 2002), which enriches the feedstock with P over time. Within individual feedstocks, increasing pyrolysis temperature was significantly correlated with higher total P contents, regardless of Mg activation level. Mass loss due to volatilization is favored at higher pyrolysis temperatures which enriches the biochar with nonvolatile P compounds as pyrolysis temperatures increase (Gunes et al. 2015; Xu et al. 2016; Zhang et al. 2017). Increasing Mg activation levels were significantly correlated with reductions in total P contents within individual feedstocks, as well as significant reductions in biochar total K contents regardless of feedstock or pyrolysis temperatures. Likewise, the contents of various micronutrients such as Zn, Mn, and Cu showed significant inverse correlations with Mg activation level irrespective of feedstock or pyrolysis temperature. The reduction of biochar nutrient content following Mg activation may be relevant as others have suggested using PL biochars as P or K fertilizers (Novak et al. 2018; Keskinen et al. 2019). Overall, our results suggest that readily soluble forms of macro and micronutrients were removed from the feedstocks during the activation process, and that the utility of the resulting biochars as a nutrient source in soil may be reduced by Mg activation.

#### 3.3.2 Proximate analysis

Biochar volatile matter (VM), fixed carbon, and ash content are presented in Additional file 1: Table S2. Decreases in biochar VM content were significantly correlated with pyrolysis temperature, regardless of PL age or Mg activation level. Since volatilization is more favorable with increasing temperature, this was expected and is consistent with previous results (Tag et al. 2016; Zhao et al. 2017). Irrespective of pyrolysis temperature or Mg activation level, decreases in the biochar fixed carbon content were significantly correlated with PL feedstock age. This is consistent with greater mineralization of organic carbon, and loss as CO_2_, within older PL (Tiquia and Tam 2002). Similarly, nonvolatile inorganic constituents are expected to be concentrated in older PL because of C loss during aging. This was reflected in our results as increasing biochar ash contents were significantly correlated with PL age, regardless of pyrolysis temperature or Mg activation level. Increases in biochar ash content were also significantly correlated with increasing pyrolysis temperatures, irrespective of PL age or Mg activation level. Higher biochar ash content with increasing pyrolysis temperature is a widely reported trend and has been attributed to the concentration of inorganics and residues of organic matter combustion at higher temperatures (Rafiq et al. 2016; Tomczyk et al. 2020).

Decreases in the concentration of biochar EP were significantly correlated with increases in the biochar ash contents. As discussed in Sect. 3.1, increasing pyrolysis temperatures favored the formation of stable P-bearing crystalline phases. Therefore, this significant relationship between biochar ash contents and EP may be reflective of the effect of pyrolysis temperature on biochar EP, as increasing biochar ash contents were also significantly correlated with increasing pyrolysis temperatures. For sorption datasets that were well described by the Langmuir model (R^2^ > 0.80, Table 1), decreases in biochar P_max_ values were significantly correlated with increases in biochar ash contents. This contrasts with the findings of others where higher P sorption by biochars was associated with higher biochar ash contents (Wang et al. 2016; Takaya et al. 2016). Our results from Sect. 3.2 suggest that P sorption was controlled by the presence of MgO in biochars derived from Mg-activated PL feedstock. Because MgO would be expected in the biochar ash content (Wang et al. 2016), this inverse relationship between P_max_ and biochar ash contents was unexpected and warrants further investigation.

#### 3.3.3 Surface area

Biochar BET surface areas (SA) are presented in Additional file 1: Table S2. Surface areas were highly variable and ranged from 3.7–245.2 m^2^ g^−1^. Surface reactivity generally increases with SA, and therefore, greater dissolution of P-bearing phases might reasonably be expected as SA increases. However, no significant correlation between biochar SA and EP concentrations was observed, indicating that SA was not a significant control on P dissolution from the biochars studied. Similarly, P_max_ values (estimated from Langmuir descriptions of sorption data with R^2^ > 0.8, Table 1) were not significantly correlated with SA. Others have partially attributed increased P sorption by Mg-activated biochars to their higher SAs compared to unactivated biochar (Zheng et al. 2020; Jung and Ahn 2016). However, our results suggest that P sorption by Mg-activated PL biochars is controlled by the formation of P sorption sites, presumably MgO, rather than any increase in biochar SA.

Increases in biochar SA were significantly correlated with the increase in pyrolysis temperature regardless of feedstock age or Mg activation level. A dependence of biochar SA on pyrolysis temperature is well reported in the literature, being attributed to the progressive decomposition of the feedstock material, formation of micropores, and volatilization of pore-blocking compounds (Tomczyk et al. 2020; Leng et al. 2021). The Mg activation of feedstocks has been previously reported to increase biochar SA. For instance, five- and 15-fold increases in the SA of biochars produced from carrot residue and *Thalia delabata*, respectively, were reported following Mg activation due to an increase in porosity (Pinto et al. 2019; Tao et al. 2019). Our results demonstrate a similar trend; when holding pyrolysis temperature constant, increasing levels of Mg activation were significantly correlated to increases in biochar SA. No significant correlation between PL feedstock age and biochar SA was observed, even when holding pyrolysis temperature and/or Mg activation level constant. Overall, our results indicate that both pyrolysis temperature and Mg activation level were more significant controls on the biochar SA than PL feedstock age.

#### 3.3.4 SEM

**Fig. 7:**
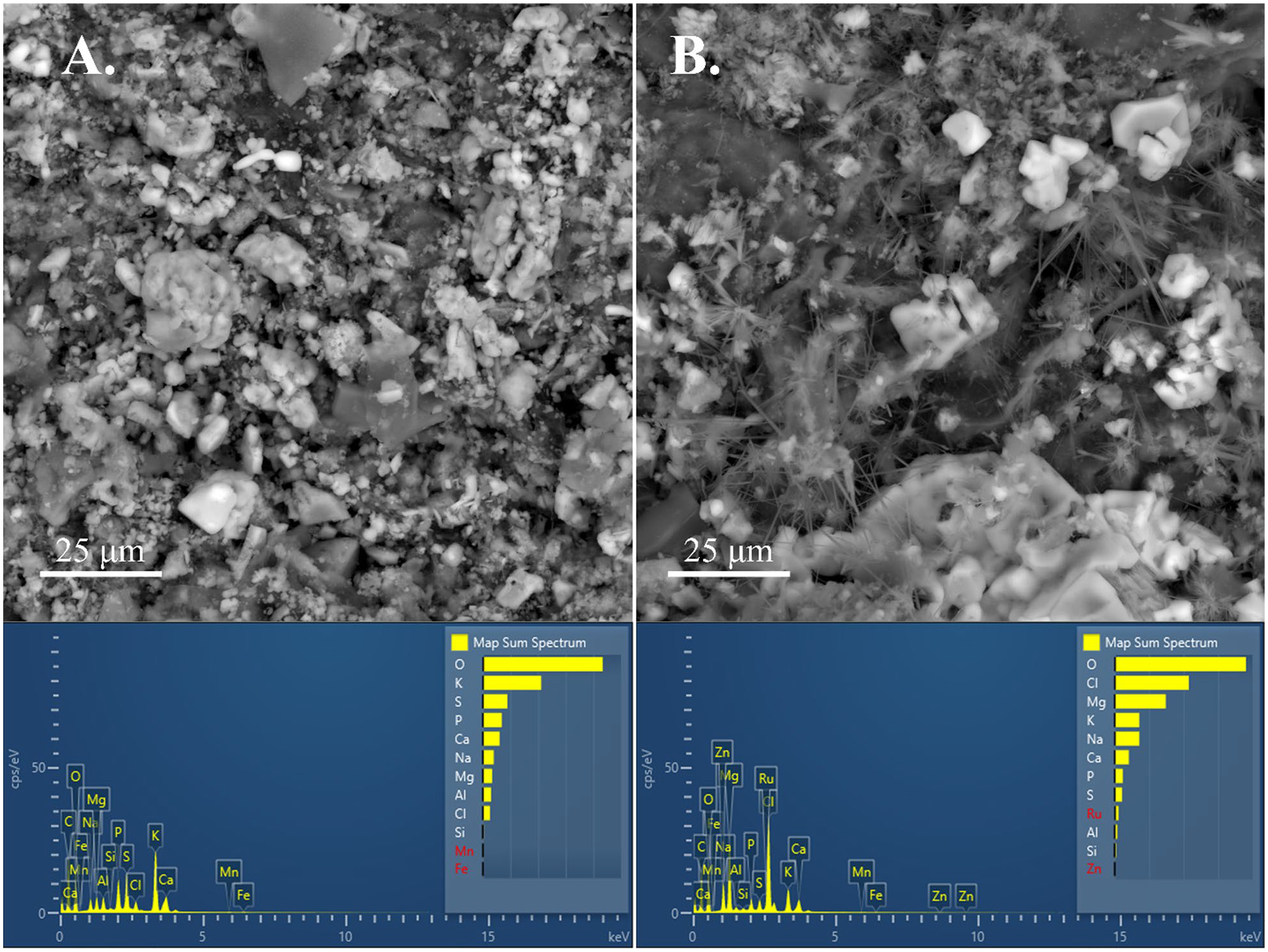
Scanning electron microscope images of the surfaces of unactivated (**A**) or 1.0 M Mg activated (**B**) biochar derived from poultry litter aged for 3–5 years and produced at 500 °C. Energy dispersive spectroscopy results for each image are shown below the SEM image

Scanning electron microscopy images of the surface of unactivated and 1.0 M Mg activated PL3 biochars produced at 500 °C are shown in Fig. 7. For the Mg activated biochar, SEM images show an abundance of needle-like crystals on the biochar surface that are not present in the image of the unactivated biochar. Surface images of other biochars produced from PL3 activated with different levels of Mg and at different pyrolysis temperatures are displayed in Supplemental Figures S6–S8, and also confirm the presence of needle-like crystals. Element maps of the activated biochar surface (Fig. 7, panel “B.”) using EDS are shown in Additional file 1: Fig. S9 and indicate a high co-location of Mg and O, suggesting that the needle-like structures are MgO crystals, consistent with our XRD results (Fig. 2). Moreover, EDS spectra indicate an increased abundance of Mg in the image of the activated biochar surface as would be expected with higher levels of Mg activation. Several others reported the presence of MgO crystals on the surface of biochars produced from a wide range of Mg-activated feedstocks, consistent with our results (Chen et al. 2018; Pinto et al. 2019; Yang et al. 2019; He et al. 2022).

#### 3.3.5 Biochar pH

The pH of all biochars is shown in Additional file 1: Table S2. The measured pH was alkaline and ranged from 8.4–11.0, consistent with other manure-derived biochars (Song and Guo 2012; Liang et al. 2014). A significant positive correlation (*p* < 0.05) between biochar pH and pyrolysis temperature was observed, regardless of feedstock age or Mg activation level. Higher biochar pH with increasing pyrolysis temperature is a well-reported trend in the literature and has been ascribed to a greater abundance of carbonates and alkali salts (Song and Guo 2012; Ding et al. 2014; Tomczyk et al. 2020). No other significant correlation (*p* > 0.05) was observed between biochar pH and feedstock age, Mg activation level, or their interactions, indicating that these production variables did not control the final biochar pH. Likewise, no significant correlations were observed between biochar pH and EP or P_max_, demonstrating that biochar pH was not a significant control on P release or sorption.

## 4 Conclusion

Biochars were produced from PL aged 1–9 years at pyrolysis temperatures of 500–900°C and Mg activation levels of 0–1 M Mg. The amount of EP for each biochar was determined and the ability of the biochars to remove additional P from solution was evaluated. Extractable P from PL-derived biochars decreased with increasing pyrolysis temperature and Mg activation level. Lower EP at higher temperatures was attributed to the formation of stable Ca_5_(PO_4_)_3_OH and Mg_3_(PO_4_)_2_ phases, while higher Mg activation levels favored the formation of Mg_3_(PO_4_)_2_. Average P_max_ values for the biochars were not affected by PL age or pyrolysis temperature. However, significantly higher average P_max_ values were achieved with 1 M Mg activation. The abundance of MgO was favored with increased Mg loading and likely accounted for higher P sorption by the 1 M Mg-activated biochars. Our results demonstrate that Mg activation is an effective strategy for both fixing the intrinsic P content of PL-derived biochars and enabling them to sorb additional P. Specifically, Mg activation levels ≥ 0.25 M and pyrolysis temperatures ≥ 700°C should be used to fix the intrinsic P content of the PL-derived biochars. To produce biochars with the highest P sorption capacity, we recommend activating the PL feedstock with 1 M Mg prior to pyrolysis. This study confirms that Mg-activated, PL-derived biochars can remove P from solution. However, further investigations are needed to evaluate the efficacy of these biochars as soil amendments to reduce P mobility in more natural systems, or in systems where legacy P in soil is of environmental concern.

## Supplementary Material

Supplement1

## Figures and Tables

**Table 1:** Estimates of maximum sorption capacity, P_max_ (mg g^−1^), for unactivated and Mg-activated biochars produced from poultry litter aged 1-year (PL1), 3–5 years (PL3), or 7–9 years (PL7)

		Pyrolysis temperature (°C)					
		500 °C		700 °C		900 °C	
Feedstock	Mg activation (M)	P_max_ ± SE (mg g^−1^)	R^2^	P_max_ ± SE (mg g^−1^)	R^2^	P_max_ ± SE (mg g^−1^)	R^2^
PL1	0	NR^a^	–	NR^a^	–	NR^a^	–
	0.25	14.52 ± 6.58	0.27	4.10 ± 0.17	0.92	0.66 ± 0.12	0.83
	0.5	NR	–	2.39 ± 0.29	0.93	1.67 ± 0.22	0.91
	1.0	8.55 ± 0.97	0.94	7.41 ± 0.23	1.00	2.60 ± 0.25	0.96
PL3	0	NR^a^	–	0.56 ± 0.25	0.59	3.36 ± 2.12	0.25
	0.25	NR^a^	–	^a^NR	–	1.19 ± 0.31	0.67
	0.5	NR^a^	–	0.38 ± 0.11	0.60	5.33 ± 0.60	0.94
	1.0	6.32 ± 0.32	0.99	7.43 ± 0.83	0.94	10.35 ± 1.45	0.90
PL7	0	NR^a^	–	NR^a^	–	NR^a^	–
	0.25	NR^a^	–	1.17 ± 1.03	0.01	0.62 ± 0.06	0.96
	0.5	134.41 ± 133.98	0.00	0.95 ± 0.19	0.79	4.25 ± 0.21	0.85
	1.0	4.90 ± 0.61	0.92	2.84 ± 0.40	0.90	5.41 ± 1.85	0.96

Estimates of P_max_ were obtained from Langmuir model descriptions of observed isotherm data

a *NR* Net P release

**Table 2:** Reported maximum P sorption capacities (P_max_) of biochars produced from Mg-activated feedstocks

Feedstock	Pyrolysis temperature (°C)	Mg:Feedstock (mol g^−1^)	P_max_ (mg g^−1^)	Mg Efficiency^a^ (mg P mol^−1^ Mg)	References
3-Year Aged PL	900	5.0 × 10^−3^	10.4	2.1 × 10^3^	Present study
Corn Stalk	450	1.4 × 10^−3^	73.3	5.1 × 10^4^	He et al. (2022)
Cow Dung	600	2.0 × 10^−3^	30.0	1.5 × 10^4^	Chen et al. (2018)
Cypress Sawdust	600	9.8 × 10^−3^	66.7	6.8 × 10^3^	Haddad et al. (2018)
Dried Carrot Powder	400	1.6 × 10^−2^	138	8.6 × 10^3^	Pinto et al. (2019)
Bamboo	600	2.0 × 10^−2^	370	1.9 × 10^4^	Jiang et al. (2018)
Ground Coffee Waste	500	3.0 × 10^−2^	56.0	1.9 × 10^3^	Shin et al. (2020)
Poultry Manure	350	3.3 × 10^−2^	251	7.6 × 10^3^	Novais et al. (2018)
Sugarcane Straw	350	3.3 × 10^−2^	17.7	5.4 × 10^2^	Novais et al. (2018)

a mg of P sorption achieved per mol of Mg used in the activation process

## Data Availability

All data are publicly available in the Dryad Data Repository.

## Highlights

- Magnesium activation of poultry litter biochar reduced extractable phosphorus and allowed for additional phosphorus sorption.
- Extractable phosphorus was reduced via the formation of solid Mg_3_(PO_4_)_2_ and Ca_5_(PO_4_)_3_OH phases.
- Phosphorus sorption capacities were the highest for biochars produced from 1 M Mg activated poultry litter.
