# Supplementary material for: Magnesium activation affects the properties and phosphate sorption capacity of poultry litter biochar
Source: Biochar. Author manuscript; Available in PMC 2024 Oct 7. (PMC10805231; doi:10.1007/s42773-023-00263-5)
Supplement: Supplement1 [file NIHMS1951534-supplement-Supplement1.docx]

**Magnesium activation affects the properties and phosphate sorption capacity of poultry litter biochar**

The Additional file 1 contains 2 tables and 3 figures.

Table S1: Best-fit Freundlich isotherm parameters for P sorption by unactived and Mg-activated biochar produced from poultry litter aged 1 year (PL1), 3-5 years (PL3), or 7-9 years (PL7)

| Feedstock | Mg Activation (M) |  | Pyrolysis Temperature | | | | | | | | | | |
| --- | --- | --- | --- | --- | --- | --- | --- | --- | --- | --- | --- | --- | --- |
|  |  |  | 500°C | | |  | 700°C | | |  | 900°C | | |
|  |  |  | K_f_  (mg^1-n^ L^n^ g^-1^) | *n* | R^2^ |  | K_f_  (mg^1-n^ L^n^ g^-1^) | n | R^2^ |  | K_f_  (mg^1-n^ L^n^ g^-1^) | n | R^2^ |
|  |  |  |  |  |  |  |  |  |  |  |  |  |  |
| PL1 | 0 |  | NR | -- | -- |  | NR | -- | -- |  | NR | -- | -- |
|  | 0.25 |  | NR | -- | -- |  | 6.65 | -0.23 | 0.64 |  | 2.02 | -0.12 | 0.47 |
|  | 0.5 |  | NR | -- | -- |  | 4.97 | -0.03 | 0.01 |  | 2.75 | -0.06 | 0.06 |
|  | 1.0 |  | 7.10 | 0.09 | 0.42 |  | 4.50 | 0.11 | 0.84 |  | 3.02 | -0.01 | 0.00 |
| PL3 | 0 |  | NR | -- | -- |  | NR | -- | -- |  | NR | -- | -- |
|  | 0.25 |  | NR | -- | -- |  | 11.16 | -0.61 | 0.41 |  | 4.83 | -0.10 | 0.47 |
|  | 0.5 |  | NR | -- | -- |  | 6.02 | -0.26 | 0.57 |  | 4.79 | 0.06 | 0.29 |
|  | 1.0 |  | 6.29 | 0.03 | 0.16 |  | 5.47 | 0.06 | 0.49 |  | 2.82 | 0.28 | 0.76 |
| PL7 | 0 |  | NR | -- | -- |  | NR | -- | -- |  | NR | -- | -- |
|  | 0.25 |  | NR | -- | -- |  | 6.17 | -0.31 | 0.99 |  | 3.12 | -0.21 | 0.41 |
|  | 0.5 |  | 1.34 | 0.06 | 0.84 |  | 7.10 | -0.28 | 0.52 |  | 4.04 | -0.20 | 0.53 |
|  | 1.0 |  | 5.01 | 0.02 | 0.07 |  | 5.23 | -0.04 | 0.11 |  | 2.59 | 0.03 | 0.03 |

Table S2: Total macro- and micronutrient content, average (n=3) values of volatile matter (VM), fixed carbon (Fixed), and ash content, and average (n=2) surface area of biochars derived from poultry litter aged 1-year (PL1), 3-5-years (PL3), and 7-9-years (PL7) as a function of pyrolysis temperature (PT) and Mg-activation level (Mg Trt.).

|  |  |  | Macro-and Micronutrients | | | | | | | |  | Proximate Analysis | | |  |  |
| --- | --- | --- | --- | --- | --- | --- | --- | --- | --- | --- | --- | --- | --- | --- | --- | --- |
| Feedstock | PT (ºC) | Mg Trt. (M) | P (%) | Mg (%) | Ca (%) | K (%) | Fe (%) | Zn  (mg kg^-1^) | Mn (%) | Cu (%) |  | VM (%) | Fixed (%) | Ash (%) | Surface Area  (m^2^ g^-1^) | pH |
|  |  |  |  |  |  |  |  |  |  |  |  |  |  |  |  |  |
| PL1 | 500 | 0 | 2.94 | 1.20 | 4.52 | 5.09 | 0.212 | 1090 | 0.129 | 0.128 |  | 21.0 | 24.7 | 54.3 | 4.32 | 8.97 |
|  |  | 0.25 | 3.16 | 4.51 | 4.86 | 2.53 | 0.377 | 585 | 0.146 | 0.135 |  | 26.1 | 23.3 | 50.5 | 5.66 | 8.41 |
|  |  | 0.5 | 3.02 | 5.87 | 4.82 | 1.28 | 0.353 | 62.3 | 0.131 | 0.113 |  | 27.9 | 25.9 | 46.2 | 10.5 | 9.63 |
|  |  | 1.0 | 2.93 | 6.26 | 4.47 | 1.74 | 0.320 | 94.5 | 0.117 | 0.0934 |  | 25.2 | 21.3 | 53.5 | 18.4 | 9.96 |
|  | 700 | 0 | 2.86 | 1.13 | 4.90 | 4.99 | 0.318 | 951 | 0.120 | 0.107 |  | 18.1 | 21.6 | 60.3 | 9.80 | 10.56 |
|  |  | 0.25 | 2.87 | 3.98 | 4.68 | 2.51 | 0.315 | 1220 | 0.133 | 0.132 |  | 9.04 | 30.1 | 60.9 | 97.8 | 9.57 |
|  |  | 0.5 | 2.83 | 6.51 | 4.22 | 2.63 | 0.294 | 1130 | 0.132 | 0.129 |  | 8.48 | 30.1 | 61.4 | 67.8 | 9.68 |
|  |  | 1.0 | 2.52 | 11.0 | 3.63 | 2.39 | 0.291 | 866 | 0.115 | 0.111 |  | 11.4 | 29.3 | 59.2 | 45.8 | 9.89 |
|  | 900 | 0 | 3.90 | 1.77 | 5.65 | 6.57 | 0.357 | 1010 | 0.184 | 0.191 |  | 13.0 | 18.1 | 68.9 | 104 | 10.46 |
|  |  | 0.25 | 2.73 | 3.95 | 3.83 | 2.38 | 0.252 | 1190 | 0.127 | 0.126 |  | 7.94 | 24.1 | 68.0 | 222 | 9.27 |
|  |  | 0.5 | 2.58 | 5.86 | 3.68 | 2.26 | 0.248 | 1160 | 0.118 | 0.121 |  | 5.85 | 27.7 | 66.5 | 245 | 9.76 |
|  |  | 1.0 | 2.22 | 9.11 | 3.20 | 2.04 | 0.242 | 1020 | 0.100 | 0.101 |  | 6.59 | 26.9 | 66.5 | 178 | 9.51 |
| PL2 | 500 | 0 | 3.90 | 1.61 | 3.98 | 6.94 | 0.165 | 1230 | 0.166 | 0.0925 |  | 32.5 | 29.0 | 38.5 | 3.72 | 8.90 |
|  |  | 0.25 | 3.12 | 4.55 | 3.40 | 3.18 | 0.193 | 958 | 0.132 | 0.0604 |  | 24.7 | 33.3 | 42.0 | 7.51 | 8.56 |
|  |  | 0.5 | 2.86 | 6.69 | 3.30 | 2.86 | 0.162 | 904 | 0.119 | 0.0546 |  | 28.3 | 31.6 | 40.1 | 6.84 | 9.52 |
|  |  | 1.0 | 2.57 | 10.7 | 2.92 | 2.63 | 0.146 | 835 | 0.105 | 0.0469 |  | 26.2 | 26.0 | 47.8 | 26.0 | 9.86 |
|  | 700 | 0 | 3.77 | 1.55 | 3.65 | 7.30 | 0.146 | 1090 | 0.150 | 0.0769 |  | 17.9 | 34.4 | 47.7 | 3.90 | 10.12 |
|  |  | 0.25 | 3.52 | 5.01 | 3.97 | 3.38 | 0.222 | 1030 | 0.150 | 0.0674 |  | 11.8 | 37.1 | 51.1 | 36.3 | 9.55 |
|  |  | 0.5 | 3.22 | 7.42 | 3.76 | 3.19 | 0.325 | 902 | 0.133 | 0.0640 |  | 13.9 | 34.3 | 51.8 | 44.4 | 9.66 |
|  |  | 1.0 | 3.09 | 12.0 | 3.81 | 3.00 | 0.202 | 804 | 0.120 | 0.0571 |  | 13.1 | 30.5 | 56.4 | 83.3 | 9.97 |
|  | 900 | 0 | 3.99 | 1.68 | 4.87 | 7.00 | 0.192 | 743 | 0.160 | 0.0801 |  | 18.2 | 29.6 | 52.2 | 75.9 | 10.77 |
|  |  | 0.25 | 3.92 | 5.54 | 4.85 | 3.78 | 0.254 | 426 | 0.162 | 0.0611 |  | 9.18 | 31.4 | 59.4 | 245 | 9.04 |
|  |  | 0.5 | 4.00 | 9.16 | 4.77 | 2.83 | 0.244 | 136 | 0.146 | 0.0502 |  | 10.4 | 30.1 | 59.5 | 243 | 9.40 |
|  |  | 1.0 | 3.37 | 13.2 | 4.01 | 2.29 | 0.223 | 116 | 0.122 | 0.0410 |  | 10.0 | 29.3 | 60.7 | 236 | 9.73 |
| PL3 | 500 | 0 | 3.65 | 1.47 | 4.60 | 6.04 | 0.183 | 1560 | 0.147 | 0.0970 |  | 22.9 | 13.2 | 63.8 | 5.52 | 9.30 |
|  |  | 0.25 | 3.47 | 3.53 | 5.29 | 2.97 | 0.763 | 1550 | 0.151 | 0.0970 |  | 23.2 | 15.8 | 61.0 | 5.73 | 8.85 |
|  |  | 0.5 | 4.02 | 5.86 | 5.22 | 2.54 | 0.327 | 1780 | 0.169 | 0.108 |  | 29.2 | 10.6 | 60.1 | 10.9 | 9.63 |
|  |  | 1.0 | 3.55 | 7.12 | 4.90 | 1.88 | 0.336 | 1530 | 0.146 | 0.0957 |  | 24.3 | 5.86 | 69.9 | 9.41 | 9.51 |
|  | 700 | 0 | 3.91 | 1.43 | 5.55 | 6.42 | 0.243 | 1540 | 0.138 | 0.110 |  | 21.9 | 8.29 | 69.8 | 7.84 | 10.41 |
|  |  | 0.25 | 4.14 | 3.88 | 6.02 | 3.30 | 0.470 | 1810 | 0.171 | 0.110 |  | 22.4 | 7.28 | 70.3 | 23.8 | 9.87 |
|  |  | 0.5 | 4.34 | 5.96 | 5.96 | 2.67 | 0.393 | 1830 | 0.177 | 0.111 |  | 17.5 | 17.3 | 65.2 | 20.3 | 9.75 |
|  |  | 1.0 | 4.22 | 8.73 | 5.74 | 2.40 | 0.388 | 1440 | 0.174 | 0.116 |  | 19.2 | 16.7 | 64.1 | 13.4 | 9.82 |
|  | 900 | 0 | 3.83 | 1.75 | 4.99 | 6.72 | 0.988 | 1130 | 0.183 | 0.129 |  | 24.5 | 2.67 | 72.8 | 52.3 | 10.97 |
|  |  | 0.25 | 4.09 | 3.88 | 6.29 | 2.81 | 0.522 | 1330 | 0.175 | 0.109 |  | 9.76 | 10.5 | 79.7 | 80.8 | 9.79 |
|  |  | 0.5 | 4.04 | 5.20 | 6.09 | 1.82 | 0.488 | 486 | 0.159 | 0.0893 |  | 9.76 | 20.3 | 70.0 | 89.9 | 8.93 |
|  |  | 1.0 | 4.39 | 8.54 | 6.33 | 1.75 | 0.488 | 146 | 0.158 | 0.0798 |  | 8.67 | 17.6 | 73.8 | 99.7 | 9.47 |
|  |  |  | ------------------------------------------------------------------------------------------ p < 0.05* -------------------------------------------------------------------------------- | | | | | | | | | | | | | |
| Feedstock | | | Y | -- | Y | -- | Y | -- | Y | -- |  | -- | Y | Y | -- | -- |
| PT | | | -- | -- | -- | -- | -- | -- | -- | -- |  | Y | -- | Y | Y | Y |
| Mg Trt. | | | -- | Y | -- | Y | -- | Y | Y | Y |  | -- | -- | -- | -- | -- |
| Feedstock x PT | | | Y | -- | Y | -- | Y | -- | Y | -- |  | -- | Y | Y | -- | -- |
| Feedstock x Mg Trt. | | | Y | Y | Y | Y | -- | -- | -- | -- |  | -- | -- | -- | -- | -- |
| PT x Mg Trt. | | | -- | Y | -- | Y | -- | Y | Y | Y |  | Y | -- | -- | Y | -- |
| Feedstock x PT x Mg Trt. | | | Y | Y | Y | Y | -- | -- | -- | -- |  | -- | -- | Y | -- | -- |

* “Y” indicates p-values <0.05 for slope estimates between production variables (feedstock, pyrolysis temperature, Mg-activation level, and their interactions) and measured properties of the biochars.

**Figures**

Figure S1: Sorbed concentrations of P by biochars produced at 700° C from poultry litter aged 1 year (PL1), 3-5 years (PL3), and 7-9 years (PL7). Initial pH of the solution was 6.0 and the initial P concentration was 75 mg L^-1^. Error bars are the 95% confidence interval. For each feedstock, Tukey pairwise comparisons (p<0.05) were conducted for each reaction time. No significant difference in sorbed P was observed for any reaction time, indicating that equilibrium was achieved following 24 hours.

Figure S2: Extractable P from biochars produced from poultry litter aged for 1 year (PL1), 3-5 years (PL3), or 7-9 years (PL7) at 500, 700, or 900°C and activated with 0-1 M Mg. Error bars are the 95% confidence interval. Within each pyrolysis temperature and Mg activation level, different letters indicate significant differences at the p<0.05 level according to a Tukey pairwise comparison test.

Figure S3: X-ray diffraction patterns for Mg-activated (0-1.0 M Mg) biochars produced from poultry litter aged 1-year (PL1), 3-5 years (PL3), and 7-9 years (PL7) at pyrolysis temperatures of 500, 700, and 900°C. Characteristic peaks are identified as: quartz (1), sylvite (2), MgO (3), Mg_3_(PO_4_)_2_ (4), Ca_5_(PO_4_)_3_OH (5).

Figure S4: Solution concentrations at the end of pH-dependent sorption experiments (1 M Mg activated biochars, 75 mg L^-1^ initial P concentration). Error bars are the 95% confidence interval. For each biochar, different letters indicate significant differences at the p<0.05 level according to a Tukey pairwise comparison test.

Figure S5: Observed data and best-fit Langmuir isotherms for P sorption by each 0-0.5 M Mg-activated biochars produced from poultry litter aged 1-year (PL1), 3-5 years (PL3), or 7-9 years (PL7) pyrolyzed at 500, 700, and 900°C. Error bars are standard errors of the mean.

**
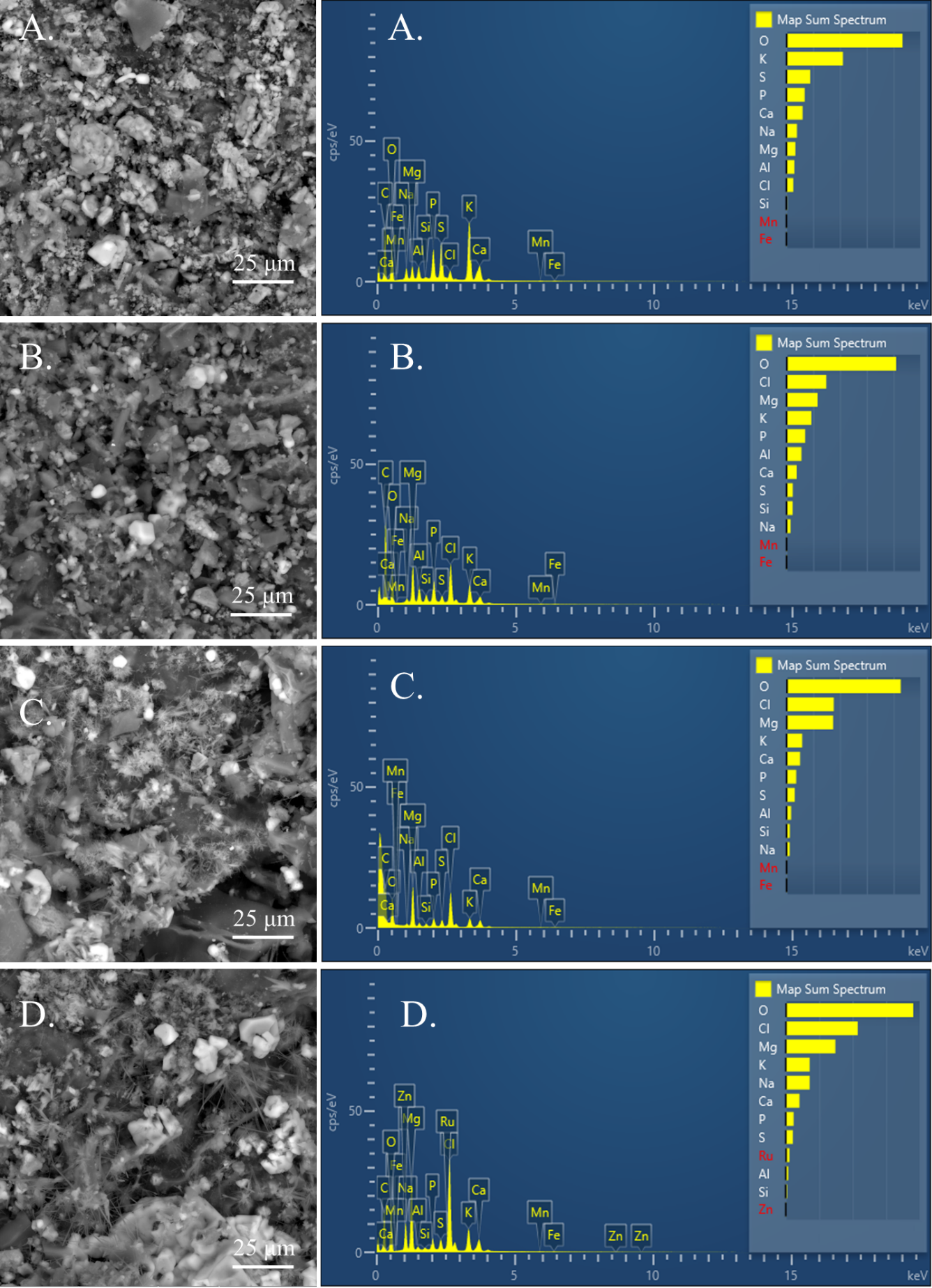
**

Figure S6: Scanning electron microscopy images and energy dispersive spectra for biochars derived from poultry litter aged 3-5 years activated with 0 M (A.), 0.25 M (B.), 0.5 M (C.), and 1.0 M (D.) Mg, produced at 500°C.


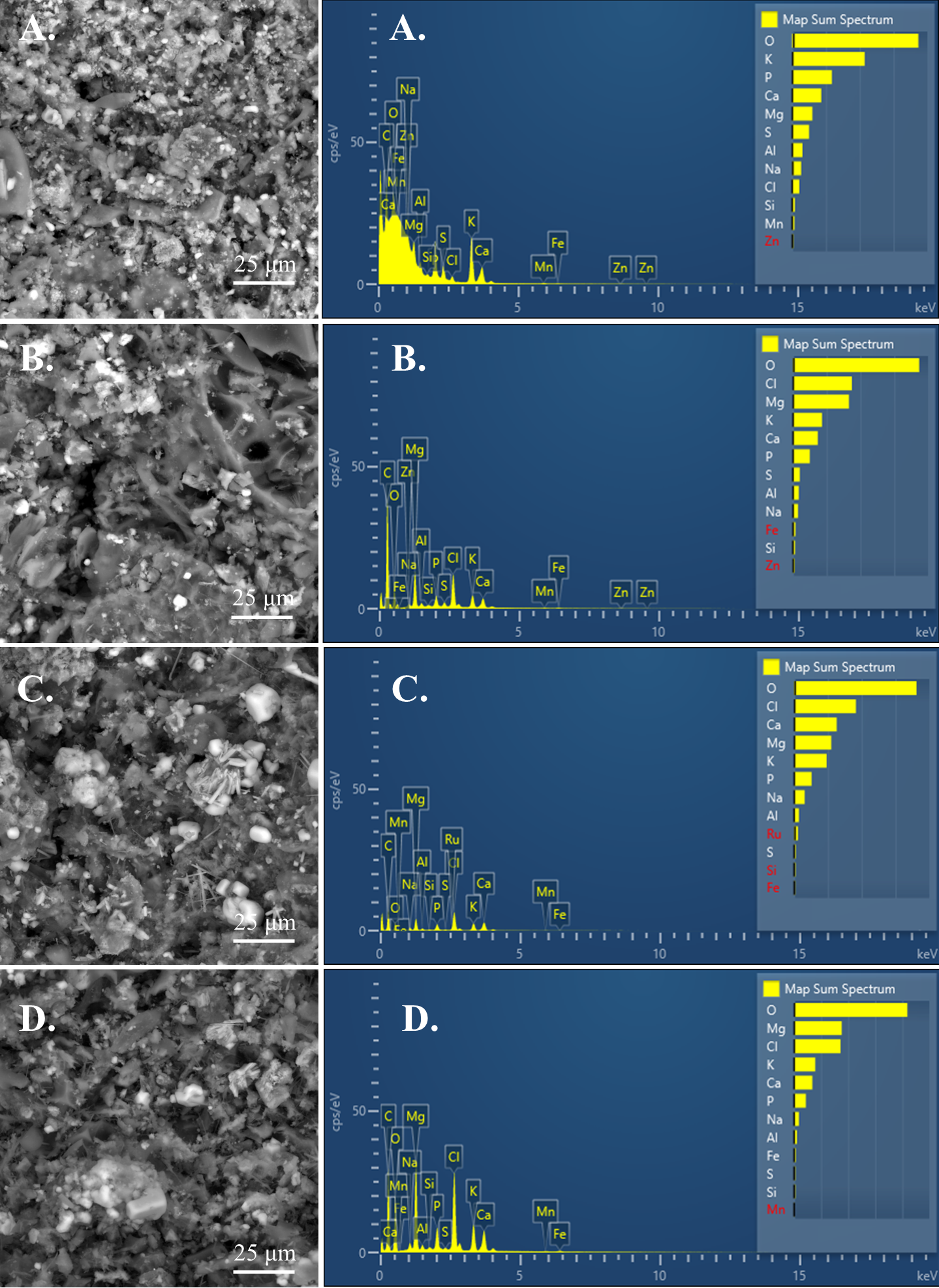


Figure S7: Scanning electron microscopy images and energy dispersive spectra for biochars derived from poultry litter aged 3-5 years activated with 0 M (A.), 0.25 M (B.), 0.5 M (C.), and 1.0 M (D.) Mg, produced at 700°C.


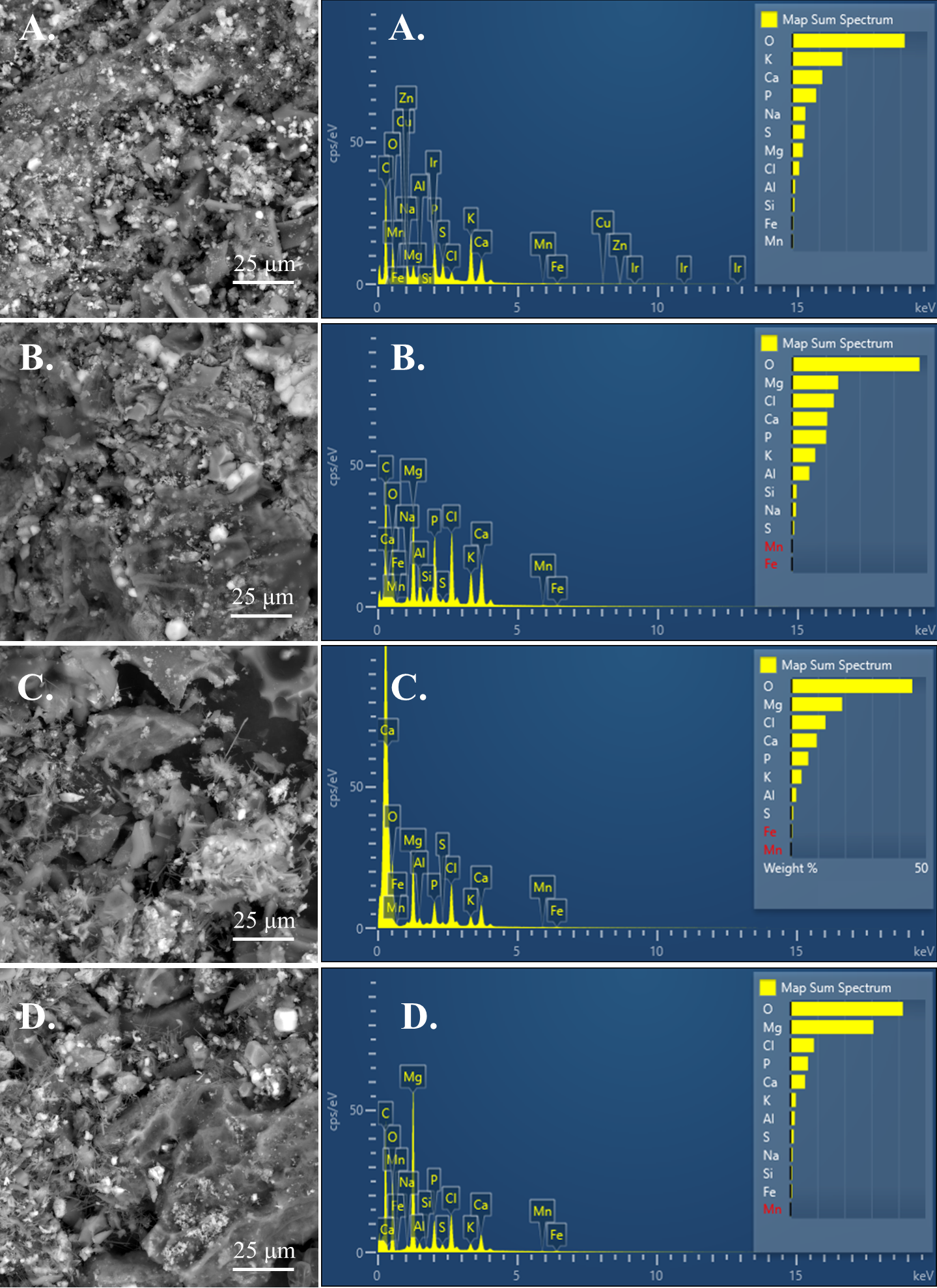


Figure S8: Scanning electron microscopy images and energy dispersive spectra for biochars derived from poultry litter aged 3-5 years activated with 0 M (A.), 0.25 M (B.), 0.5 M (C.), and 1.0 M (D.) Mg, produced at 700°C.


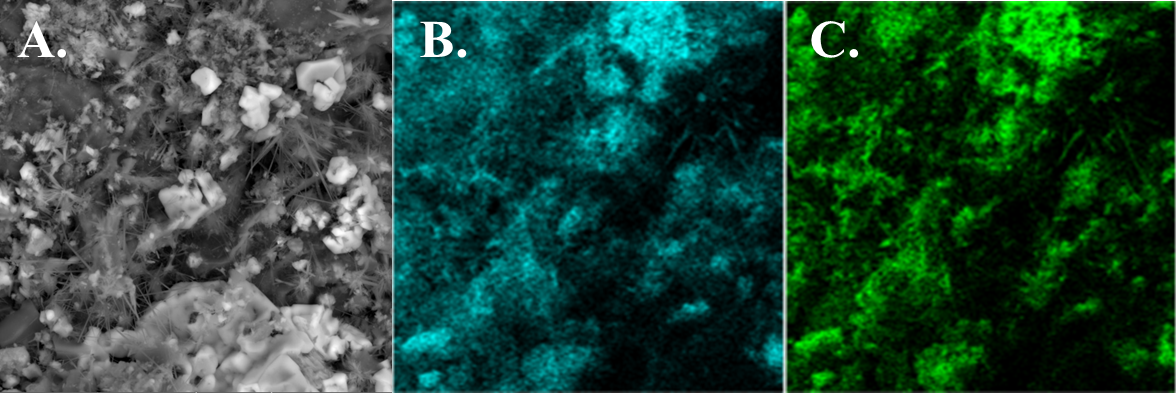


Figure S9: Scanning electron microscope image (A.) and energy dispersive spectroscopy mapping of Mg (B.) and O (C.) of biochar derived from 1.0 M Mg activated poultry litter aged for 3-5 years, produced at a pyrolysis temperature of 500°C.
